# Perceived health, quality of life and happiness among older professional artisans in a UNESCO Creative City of crafts and folk art

**DOI:** 10.3389/fpsyg.2025.1536447

**Published:** 2025-05-09

**Authors:** Sandra Igreja, Soraia Teles, Constança Paúl

**Affiliations:** RISE-Health, Department of Behavioral Sciences, School of Medicine and Biomedical Sciences, University of Porto (ICBAS-UP), Porto, Portugal

**Keywords:** older adults, crafts, health, quality of life, happiness, active ageing

## Abstract

Globally, it is a priority to understand how to improve health, quality of life, and happiness in a long-lived and rapidly aging population. Evidence on the health outcomes of working in later life is mixed, with some studies suggesting it benefits mental health by preserving identity, status, social participation, and a sense of purpose, while others highlight potential adverse effects. Research on aging at work has seldom focused on older adults professionally engaged in artistic activities, particularly across different craft sectors, in contrast to the extensive research on art-based interventions aimed at promoting well-being among older individuals. This study aims to describe the perceptions of health, quality of life, and happiness among older professional artisans from a UNESCO Creative City of Crafts and Folk Art in Portugal, and examine the associations with sociodemographic and professional variables. A cross-sectional study was conducted, involving primary data collection through a survey administered in the participants’ (*N* = 55) work environments. Eligible participants were individuals aged 55 or older, living in the community, and working in various craft sectors. The artisans completed the WHOQOL-BREF scale, were assessed for depressive symptoms with the PHQ-8 scale, and were inquired on happiness with life using an item from the European Survey on Aging Protocol (ESAP). Most artisans perceived their overall quality of life as ‘good’, with the psychological domain receiving the highest score (M = 84.92; SD = 10.98). Most artisans (56.4%) also reported feeling ‘very happy’ and being satisfied or very satisfied with their health (60%). Age was positively correlated with happiness (*p* = 0.020). A significant difference was observed in the WHOQOL-BREF environment domain between craft sectors (*p* = 0.023). An association was observed between different craft sectors and their perceived benefits, particularly regarding health, quality of life, and well-being. This study contributes to aging research by focusing on professional activity in the craft and folk art sector, highlighting the intersection of aging, work, and artistic engagement, and offering insights for policy development to support aging craftspeople and promote traditional crafts.

## 1 Introduction

Mental health in older adults can be improved through the promotion of active and healthy aging (World Health Organization, 2023). The WHO defines mental health as a state of well-being in which an individual is aware of their abilities, can manage the normal stresses of daily life, work productively and fruitfully, and is able to contribute to their community. Emotional well-being and mental health are as important in old age as at any other stage of the life cycle (Santos, 2020). The primary aim of active and healthy aging is well-being, a holistic concept that encompasses all the elements and components of life valued by the individual (Benavente, 2020).

Intergovernmental organizations, such as the United Nations, have encouraged countries to use well-being indicators in making important policy decisions, in addition to traditional economic indicators, recognizing the limitations of this approach (Kim et al., 2021; United Nations, 2023). The literature supports the planning of healthy policies that consider the perspectives of older adults, providing resources and activities related to health, participation, and safety, thereby contributing to a better quality of life (Wongsala et al., 2021).

In this sense, quality of life is characterized by its multidimensionality (Diener and Suh, 1997; Fernández-Ballesteros, 1998; Canavarro, 2010) and highlight the need to consider aspects valued by older adults for overall well-being, such as health, life satisfaction, and psychological well-being, as well as satisfaction with the social and physical environment in which they are situated (Paúl, 2017). The quality of life of older adults depends on the context or circumstances in which they live. The ingredients of quality of life are dependent on lifestyle (Fernández-Ballesteros, 1998).

Individual well-being has emerged as a new political ambition for old age, and the ability to remain active is a condition for that well-being (Baeriswyl and Oris, 2021). Happiness and well-being have been associated with favorable profiles of health-related biomarkers and go beyond reduced health issues and mortality; they are linked to a higher quality of life (Ryff, 1989; Ryff, 1995; Diener et al., 1998; Diener, 2000; Novo, 2000; Steptoe, 2019; Becker and Trautmann, 2022; Kokubun et al., 2022).

Furthermore, aging requires that the role of older adults extend beyond just physical and occupational activities to include, among other aspects, participation in social, economic, and cultural processes. Productive activities are those that hold significant meaning for the individual and create social value, whether they are compensated or not (Vega-Tinoco et al., 2022). Professional activity assumes special importance. Work is not only associated with income; it should also be seen as a source of personal achievement, contributing to the maintenance of one’s status and identity outside the family context (Quartilho, 2010). From these conclusions arises the proposal to provide the older population with opportunities for aging through broader professional participation (Park and Lee, 2022). Education and employment consistently appear as primary determinants of health and economic outcomes across all stages of life, including in older age (United Nations, 2023).

Research on active aging has expanded over the past few decades. However, the body of studies on activities related to crafts performed by older adults, in natural contexts, and not resulting from therapeutic interventions, is limited and rarely focuses on crafts and folk art, involving both male and female artisans (Tzanidaki and Reynolds, 2011; Liddle et al., 2013; Noice et al., 2014; Pöllänen and Weissmann-Hanski, 2020; Chacur et al., 2022). This article explores the perception of health, quality of life, and happiness in a sample of older professional artisans from Barcelos, a UNESCO Creative City of Crafts and Folk Art in Portugal. The study provides a unique analysis of this context, involving both men and women, as well as artisans from various craft sectors within the region. It also examines the association between sociodemographic characteristics, professional sector variables, and health, quality of life, and happiness.

In the early 1990s, the concept of active aging began to develop, offering a policy framework that emphasizes the connection between activity, health, independence, and aging well (Paúl and Lopes, 2016). At the beginning of the 21st century, the world summit on population endorsed active aging—“the process of optimizing opportunities for health, participation and security in order to enhance quality of life as people age” as the primary objective of social and health policies for older adults (World Health Organization, 2002, p. 12). Active aging remains a complex construct, and studies have shown that, globally, subjective and objective health and functionality are its main components. By staying active, older adults seem to overcome difficulties and remain highly motivated to participate in the social world and engage in healthy behaviors that enhance quality of life throughout the aging process (Paúl et al., 2012).

The central rhetoric of active aging emphasizes the autonomy and capacity of older adults to engage in meaningful social actions, contrasting with disengagement and opposing the culture of early retirement. It advocates for the removal of age-related barriers to labor market participation and the extension of active careers to delay or prevent reductions in social and institutional engagement. This approach promotes healthy lifestyles and overall quality of life, positioning continuous participation in society as a key component of successful aging (Cabral et al., 2013; Paúl and Lopes, 2016).

Active aging is a powerful discourse because it aligns more closely with the actual capabilities of older adults. It is important to recognize that there are multiple pathways for older individuals to age actively. Active aging policies should focus on overall life participation, rather than limiting the concept to economic activity or highly physical activities (Boudiny, 2013). Over the decades, a substantial amount of research has accumulated on individual, work-related, team, organizational, non-work, and social factors that contribute to active aging in the workplace. Evidence-based implications for organizations can be derived from this research (Zacher et al., 2018).

The concept of active aging was originally based on three pillars: participation, health, and security. In 2015, this approach was reviewed and expanded to include lifelong learning, which strengthens the original pillars and recognizes information as an essential element for active aging (Bárrios, 2015; Paúl and Lopes, 2016). The WHO model of active aging was revised to emphasize the optimization of four key pillars, providing older individuals with a policy framework to maximize their well-being potential, which, in turn, may contribute to greater longevity (Hijas-Gómez et al., 2020). On the other hand, healthy aging, as defined by the WHO, is “the process of developing and maintaining functional ability that enables well-being in older age,” and it replaced the emphasis previously placed on active aging, a policy framework developed in 2002 (World Health Organization, 2020; United Nations, 2023). Healthy aging emphasizes the importance of developing and maintaining functional abilities, recognizing that these depend on each individual’s intrinsic capacity, the surrounding environment, and the interactions between both (United Nations, 2023).

Healthy aging has become the focus of the World Health Organization, as reflected in the designation of the Decade of Healthy Aging (2021–2030). However, an emphasis on the need for action across multiple sectors endures, to ensure that older adults continue to be a resource for their families, communities, and economies (United Nations, 2020, 2023). Governments should remove barriers to older adults’ participation in the workforce while supporting their learning and skills development throughout their lifetime (United Nations, 2023).

In this regard, paying closer attention to the potential of older adults and their contributions to society involves shifting away from the dominant view of them as mere beneficiaries of innovation to a perspective that recognizes innovation created by older adults. This includes adopting a more positive mindset towards aging, where the voices of older individuals are heard (Noack and Federwisch, 2020).

There is a growing interest in understanding and exploring how the arts contribute to health and quality of life in older adults. There is a need to consider contributions for improving life, as well as the quality and best practices of various art-based modalities, in order to understand the impact of the arts on older individuals (Fraser et al., 2015; Archibald and Kitson, 2020). The therapeutic use of the arts has been documented since antiquity. For centuries, artists, philosophers, doctors, and other professionals have highlighted the specific benefits of the arts for health and well-being. Interest in using the arts to influence health grew substantially in the 20th century (Chapline and Johnson, 2016). In this context, the literature has identified improvements in health, well-being and quality of life in older adults through active participation and engagement in the arts and arts-based programs (Gale et al., 2012; Noice et al., 2014; Curtis et al., 2018; Ho et al., 2019; Lewis et al., 2021; Pesata et al., 2022). Artistic activities, by combining cognitive flexibility, creativity, interpersonal behaviors, generosity, and cooperation, can be particularly effective in reducing symptoms in those with depression (Dunphy et al., 2019; Fancourt and Ali, 2019). Numerous benefits have been described in the literature regarding the impact of engaging in the arts on mental health (Van Lith et al., 2013; Williams et al., 2019; Burns and Van Der Meer, 2021; Nan et al., 2021; Jenabi et al., 2022; Keisari et al., 2022). The body of research on activities related to arts by older adults has rarely focused on crafts and folk arts (Fraser et al., 2015; Chacur et al., 2022), not only as an hobby but also as an economic activity.

This study focuses on older professional artisans working in crafts and folk art across various sectors of a UNESCO Creative City (Município de Barcelos, 2023; UNESCO, 2024). There is a growing concern about the role of employment in the subjective well-being of older populations (Chang and Yen, 2011). Craft has been classified as one of the art forms in research on arts and health (Davies et al., 2012). Improving and maintaining the well-being of older individuals while reducing healthcare costs are compelling goals for promoting successful aging worldwide. A sense of meaning may be a promising target for promoting successful aging among older adults (Zhang et al., 2018).

A considerable body of research demonstrates that the self is a valuable source of meaning in work (Rosso et al., 2010). Individuals craft the meaning of their work by reorganizing tasks, redefining relationships, and reframing perceptions to assign meaning, purpose, and identity to their professional activity (Wrzesniewski and Dutton, 2001). Moreover, they are active agents who seek to realize their potential and develop harmonious passions associated with their activities (Vallerand, 2012). Recent studies on the purpose of life indicate that more resilient individuals tend to have a clear sense of purpose, which is associated with higher levels of psychological well-being and happiness (Tavoosi et al., 2024). Positive associations have been found between subjective happiness and generativity (Shahen et al., 2019), and happiness was significantly predicted by engagement in the creating arts and crafting (Keyes et al., 2024). Previous studies in other activity contexts have shown that work is an important factor for quality of life, highlighting the need for further research to clarify the relationship between occupation and health-related factors, such as depression (Min and Cho, 2018).

It is important to investigate health, quality of life, and happiness in older adults, especially in light of psychological development stages. Erikson’s Theory of Psychosocial Development (1950) expands the understanding of human development across the lifespan, recognizing that psychological growth does not cease in adulthood but continues throughout life. Erikson identifies that in old age, the central conflict is between integrity and despair, where individuals must reflect on their lives and attain a sense of fulfillment and acceptance. For older adults, what matters most is their vital engagement—with people, activities, materials, ideas, and institutions—which significantly contributes to psychological well-being (Kivnick and Wells, 2014; Bugajska, 2017).

This study draws on this theory to describe a sample of older professional artisans, exploring their sociodemographic and professional profiles, as well as their perceived health, quality of life, and happiness. Furthermore, it examines the association between sociodemographic characteristics and professional sector variables with health, quality of life, and happiness.

The study investigates variables related to health status, quality of life, and happiness among older professional artisans. The literature suggests that work and continued engagement in occupational activities can enhance health and quality of life while also helping to mitigate age-related discrimination. The WHO highlights the urgent need to implement effective anti-ageism strategies to combat age-related prejudice (World Health Organization, 2021). Previous studies conducted in various occupational contexts that assessed quality of life among older adults who are employed and those who are not indicate that maintaining an occupation is a crucial factor for preserving physical and mental health in older individuals. Additionally, it contributes to autonomy, as well as cognitive and sensory abilities, with working older adults showing higher average scores across most domains, particularly in the psychological domain (Costa et al., 2018). Furthermore, the literature suggests that factors such as gender, educational level, and living arrangements influence engagement in physical activities. The environment and social interactions of older adults are critical in understanding how their contextual surroundings impact a healthy lifestyle (Parra-Rizo et al., 2022). There is also a lack of evidence regarding the quality of life of older workers and a scarcity of interventions aimed at enabling them to extend their healthy professional lives (Baxter et al., 2021).

In the context of older professional artisans, the activity provided by working in crafts and folk art may have an impact on their perceived health, quality of life, and happiness. In this context, it is important to investigate this perception among artisans, as well as the relationship between sociodemographic characteristics, professional sector variables with health, quality of life, and happiness. It is important to note that, in addition to being a less explored area in the literature, it involves a sample of professional artisans from different sectors of crafts and folk art, and includes both male and female artisans (Noice et al., 2014; Fraser et al., 2015; Chacur et al., 2022). This may provide important insights into the understanding of this group of older adults and the benefits of active aging. The results could offer valuable information for the field of aging science, as well as allow for comparisons with international research.

The domain of crafting has always been challenging to define conceptually. The production of objects and artifacts appears to follow two main directions: on one hand, the increasing production of practical tools; on the other, the development of activities with objectives that gain symbolic, ritualistic, and magical dimensions, demonstrating significant autonomy (Pereira, 2024).

Craft or artisanal products are defined by UNESCO as “Products that are produced by artisans, either completely by hand or with the help of hand-tools or even mechanical means, as long as the direct manual contribution of the artisan remains the most substantial component of the finished product… The special nature of artisanal products derives from their distinctive features, which can be utilitarian, aesthetic, artistic, creative, culturally attached, decorative, functional, traditional, religiously and socially symbolic and significant” (UNESCO Institute for Statistics, 1997).

Consequently, craft activities, besides being a mean of subsistence, can support psychological well-being and contribute to a fulfilling life in the long term, serving as a meaningful occupation for those with an interest in this area (Pöllänen and Weissmann-Hanski, 2020). People who engage in crafting often invest emotionally in these activities, and many continue to do so throughout all stages of life (Kenning, 2015).

In Barcelos (Portugal), the commitment to crafts is both evident and fascinating. The crafts are sold and appeal to the market, and there is an ongoing production where thematic and formal models are repeated and recreated by personal interpretation. The total identification with a culture inherited through tradition, the preservation of its myths, and even the repetition of its products did not hinder more original artistic creation in Barcelos (Costa, 1991, 2024).

Barcelos is a region distinguished by its extensive artisanal activities across various craft sectors, with a natural predominance in pottery and clay figurines. This region has been designated by UNESCO as a Creative City of Crafts and Folk Art. The ateliers in the area encompass not only ceramics-related arts but also other sectors of traditional crafts and folk art, including Imagery, Pottery, Embroidery and Weaving, Iron and Derivatives, Wood, Basketry and Wicker, and Contemporary Crafts. The Pottery of Barcelos (Olaria de Barcelos), the Imagery of Barcelos (Figurado de Barcelos), and the Crivo Embroidery of São Miguel da Carreira (Bordado de Crivo de São Miguel da Carreira) are certified artisanal products (Comissão Nacional da UNESCO, 2023; Município de Barcelos, 2023; UNESCO, 2024).

In addition to the fact that research on artistic activities rarely focuses on crafts and folk art, no studies have been identified that examine the perception of health status and quality of life among older professional artisans across different sectors of crafts. In light of this, the article aims to describe a sample of older professional artisans from a UNESCO Creative City of Crafts and Folk Art in Portugal (Barcelos), considering their sociodemographic and professional characteristics, their perception of health, quality of life, and happiness; and to examine the association between sociodemographic characteristics, professional sector variables with health, quality of life, and happiness.

## 2 Materials and methods

### 2.1 Study design

An observational, cross-sectional study was conducted, involving in-person primary data collection through a survey that was hetero-administered in the participants’ working environments. This approach is appropriate for this study as it allows for the description of artisans’ characteristics and their perceptions of health, quality of life, and happiness, as well as the analysis of associations between these variables.

### 2.2 Participants and recruitment

Primary data were collected from a non-probabilistic sample of older professional artisans working in Barcelos, a UNESCO Creative City of Crafts and Folk Art. Eligible participants were individuals aged 55 or older, living in the community (not in institutional care), and working in the craft sector in ateliers located in Barcelos, Portugal. If meeting the eligibility criteria, artisans from any of the following craft sectors could participate: Imagery, Pottery, Embroidery and Weaving, Iron and Derivatives, Wood, Basketry and Wicker, and Contemporary Crafts. Potential participants were identified through public platforms, including the websites of artisans with online presences and the official website of the Municipality of Barcelos, which maps the craft routes (a list of artisans, including those without an online presence, with their atelier addresses and contact details) by craft sector. A convenience sample was used, aiming to include participants who represented the various sectors of crafts in the region. Artisans were contacted, fully informed about the study, and invited to participate. Those agreeing to participate were assessed for eligibility, and subsequent data collection stages were scheduled with those who met the criteria. All participants signed consent forms to participate in the research.

Data collection was conducted in-person in the first quarter of 2024, at the participants’ ateliers and according to a previously agreed-upon schedule.

This study received a favourable opinion by the Ethics Committee of the Centro Hospitalar Universitário de Santo António, E.P.E. (CHUdSA) and the School of Medicine and Biomedical Sciences, University of Porto (ICBAS-UP) [CHUdSA/ICBAS Ethics Committee] [reference 2024/CE/P02 (P418/2023/CETI)]. The study was conducted in accordance with local legislation and institutional requirements. All participants were provided with information about the project and signed the informed consent form, which was free and fully explained, for participation in the research study.

### 2.3 Instruments

A purposefully designed questionnaire was administered to study participants to collect sociodemographic, professional, and health-related information.

Information was collected on the sociodemographic characteristics of older professional artisans, such as age, gender, marital status, variables related to education and professional training, housing arrangements, and economic situation.

Health-related information included the type of health subsystem, use of health services in the last year frequency of appointments to the health centre and diagnosed diseases.

Information collected on the professional craft activity included the craft sector, starting age, weekly hours dedicated to the activity, and whether the atelier is in the place of residence. The perceived benefits of their artistic activity were also assessed and questions to explore activities undertaken in addition to professional work, as well as the time dedicated to them.

For a comprehensive assessment of the variables under analysis, validated and widely used instruments were employed to measure the perception of quality of life (World Health Organization, 1996; Canavarro et al., 2010), assess the presence of depressive symptoms (Spitzer et al., 2006; Kroenke et al., 2009; Kroenke et al., 2010) and evaluate the perception of happiness (Fernández-Ballesteros et al., 2004), complementing the information collected through a detailed questionnaire specifically developed for this study.

To explore perceptions of quality of life and health status, the World Health Organisation Quality of Life scale WHOQOL-BREF (World Health Organization, 1996; Canavarro et al., 2010) was used. The WHOQOL-BREF consists of 26 questions, with two questions addressing the overall perception of quality of life and health, and the remaining 24 questions distributed across the four domains: physical, psychological, social relationships, and environment. Examples of representative items for each domain include: for the physical domain, “How satisfied are you with your ability to perform your daily living activities?”; for the psychological domain, “How satisfied are you with yourself?”; for the social relationships domain, “How satisfied are you with your personal relationships?”; and for the environment domain, “How satisfied are you with your access to health services?.” Responses are based on a Likert scale, with scores ranging from one to five. Higher scores for each domain indicate a better quality of life. The European Portuguese version of the WHOQOL-BREF has demonstrated strong psychometric properties, indicating the instrument’s quality for assessing quality of life in Portugal. The instrument has good internal consistency indices when considering all 26 questions that constitute the instrument (*α* = 0.92). When analyzed individually, the domains also present very acceptable Cronbach’s alphas: Social Relationships (*α* = 0.64), Environment (*α* = 0.78), Psychological (*α* = 0.84), and Physical (*α* = 0.87) (Canavarro et al., 2010).

The Patient Health Questionnaire PHQ-8 scale (Kroenke et al., 2009) was added to assess the presence of depressive symptoms. It consists of eight items, with answers summed to produce a severity score ranging from zero to twenty-four. Representative examples of items from this scale include: “Little interest or pleasure in doing things”; “Feeling down, depressed, or hopeless”; “Felling tired or having little energy.” Scores are calculated by assigning zero to three points to statements based on frequency, ranging from “never” to “almost every day.” Scores of five, ten, fifteen, and twenty represent cut-off points for mild, moderate, moderately severe, and severe depression, respectively (Spitzer et al., 2006).

The PHQ-8 demonstrates good sensitivity and specificity for detecting depressive disorders (Kroenke et al., 2010). The diagnostic algorithm or a cutoff score of ≥ 10 can be used to define current depression (Kroenke et al., 2009). Studies that have evaluated the PHQ-8, in Portugal and internationally, indicated that the PHQ-8 has a unidimensional structure with evidence of good validity and reliability (Monteiro et al., 2019; Vasconcelos-Raposo et al., 2022). The validation of the PHQ-8 in Portugal showed a good internal consistency of the instrument (α = 0.89) (Vasconcelos-Raposo et al., 2022).

Finally, a question of happiness from The European Survey on Aging Protocol (ESAP) (Fernández-Ballesteros et al., 2004) was used to assess the degree of happiness participants feel about their current life. The question, “Compared to other people and considering the balance between the good and bad events in your life, to what extent do you feel well and happy at this moment?” is answered on a scale from one to four, with one corresponding to an extremely low level of satisfaction and four to an extremely high level of satisfaction. Based on the responses, participants were classified into four categories of happiness: very happy, happy, somewhat unhappy, and very unhappy.

### 2.4 Data analysis

Descriptive statistics were calculated to characterize participants in terms of sociodemographic, professional, health-related, quality of life, and happiness appraisal variables (Tables 1–3).

**Table 1:** Describes the study variables related to participants’ sociodemographic characteristics and health conditions.

Variables	N	Descriptive statistics
Sociodemographic characteristics		
Age (years), M (SD)	55	67.49 (8.02)
Gender, *n* (%)	55	
Male		33 (60)
Marital status, *n* (%)	55	
Married		47 (85.5)
Widow(er)		5 (9.1)
Divorced		2 (3.6)
Single		1 (1.8)
Years education, M (SD)	55	6.42 (3.32)
Professional training, *n* (%)	55	
Yes		13 (23.6)
Retired, *n* (%)	55	
Yes		33 (60)
Main professional activity, crafts, *n* (%)	55	
Yes		49 (89.1)
Main source of income, *n* (%)	55	
Crafts		54 (98.2)
Pension/Retirement		32 (58.2)
Other income		6 (10.9)
Monthly income, based on the National Minimum Wage (NMW), *n* (%)	55	
Monthly income ≤ NMW		35 (63.6)
Monthly income > NMW		20 (36.4)
Health condition		
Frequency of visits to the Health Center, *n* (%)	55	
Every 1 to 3 months		11 (20)
Every 6 months		29 (52.7)
Once a year		13 (23.6)
Less than once a year		2 (3.6)
Healthcare utilization in the past year		
Primary Care Center, *n* (%)	55	
Yes		52 (94.5)
Specialty consultations, *n* (%)	55	
Yes		34 (61.8)
Emergency services, *n* (%)	55	
Yes		9 (16.4)
Hospitalization, *n* (%)	55	
Yes		4 (7.3)

**Table 2:** Describes the study variables related to participants’ professional and artistic sector.

Variables	N	Descriptive statistics
Craft sector, *n* (%)	55	
Imagery		29 (52.7)
Pottery		7 (12.7)
Wood		5 (9.1)
Iron and derivatives		4 (7.3)
Embroidery		3 (5.5)
Contemporary crafts		3 (5.5)
Weaving		2 (3.6)
Basketry and wicker		2 (3.6)
Age of entry into the craft sector, M (SD)	55	18.65 (16.10)
Atelier location at the residence, *n* (%)	55	
Yes		48 (87.3)
Hours per week of craft activity, M (SD)	55	51.24 (17.05)
Weekly hours dedicated to non-professional activities, M (SD)	55	10.05 (9.33)
Most frequently reported non-professional activities, *n* (%)	55	
Household activities and subsistence farming		25 (45.5)
Physical activity		20 (36.4)
Sociocultural activities		16 (29.1)
Perceived main benefits of involvement in the craft sector, *n* (%)	55	
Well-being		41 (74.5)
Economic		18 (32.7)
Health		13 (23.6)
Quality of life		12 (21.8)
Social status		7 (12.7)

**Table 3:** Describes objective health indicators and outcomes related to health, quality of life, and happiness.

Variables	N	Descriptive statistics
Diagnosed diseases, *n* (%)	55	
Yes		45 (81.8)
Most commonly reported diseases, *n* (%)		
Osteoarthritis		10 (18.2)
Spinal disorders		9 (16.4)
Hip diseases, knee diseases		7 (12.7)
PHQ-8 Patient Health Questionnaire, *n* (%)	55	
Without depression (< 10)		54 (98.1)
With depression (≥ 10)		1 (1.8)
WHOQOL-BREF		
Question 2: How satisfied are you with your health? *n* (%)	55	
Dissatisfied		3 (5.5)
Neither satisfied nor dissatisfied		19 (34.5)
Satisfied and very satisfied		33 (60)
Question 1: How would you rate your quality of life? *n* (%)	55	
Poor		1 (1.8)
Neither good nor poor		13 (23.6)
Good		34 (61.8)
Very good		7 (12.7)
WHOQOL-BREF, M (SD)	55	
WHOQOL-BREF, Overall Quality of Life (questions 1 and 2)		68.64 (13.58)
WHOQOL-BREF, Psychological Domain		84.92 (10.98)
WHOQOL-BREF, Social Relationships Domain		80.76 (12.61)
WHOQOL-BREF, Environment Domain		78.81 (9.48)
WHOQOL-BREF, Physical Domain		78.64 (11.91)
Question of happiness		
Level of happiness, *n* (%)	55	
Very happy		31 (56.4)
Happy		23 (41.8)
Somewhat unhappy		1 (1.8)

For the WHOQOL-BREF scale, the original scores for each domain were transformed to a scale of 0 to 100 for interpretability purposes, following scoring guidelines (World Health Organization, 1996; Canavarro et al., 2010).

Absolute and relative frequencies were used to describe the distribution of the data. Measures of central tendency (e.g., mean and median) and dispersion (e.g., standard deviation and variance) were applied to summarise and analyse the location and variability of the data.

To examine the associations between sociodemographic characteristics and professional sector variables with health, quality of life, and happiness parametric and non-parametric tests were chosen as appropriate, based on data distribution. Means and data distributions were compared using the t-test for parametric continuous variables, and the Mann–Whitney U test for non-parametric continuous or ordinal variables. Associations between categorical variables were analysed using the chi-squared test and Fisher’s exact test, as appropriate. Correlations were examined using Pearson’s correlation coefficient and Spearman’s rank correlation coefficient, depending on the nature of the data. The F-statistic was used in ANOVA to compare means across multiple groups. Statistical tests were two-tailed, and the significance level was set at 0.05 (Table 4). Data analysis was performed using SPSS version 29. The present study follows the Strengthening the Reporting of Observational Studies in Epidemiology (STROBE) statement, which provides guidelines for reporting observational studies (von Elm et al., 2007). The STROBE checklist is included in Supplementary material 1.

**Table 4:** Significant associations between sociodemographic and professional variables, objective health indicators, and outcomes of health, quality of life, and happiness.

Sociodemographic and professional variables, objective health indicators vs. health, quality of life, and happiness outcomes	*p*-value*
Age vs. Happiness	0.020^1^
Gender: Female vs. Most commonly reported diseases: Osteoarthritis	< 0.001^2^; < 0.001^3^
Gender: Female vs. Perceived main benefits of involvement in the craft sector: Quality of life	0.033^2^; 0.047^3^
Gender: Male vs. Main benefit of involvement in the craft sector: Well-being	0.032^2^
Monthly income, based on the National Minimum Wage vs. WHOQOL-BREF, overall quality of life and health (questions 1 and 2)	0.018^4^
Monthly income, based on the National Minimum Wage vs. WHOQOL-BREF, Environment Domain	0.027^5^; < 0.001^6^
Reported Illnesses / No Reported Illnesses vs. Main benefit of involvement in the craft sector: Health	0.030^2^; 0.045^3^
Reported Illnesses / No Reported Illnesses vs. WHOQOL-BREF, overall quality of life and health (questions 1 and 2)	0.017^4^
Imagery craft sector vs. Main benefit of involvement in the craft sector: Health	0.008^2^; 0.011^3^
Imagery craft sector vs. Main benefit of involvement in the craft sector: Quality of life	0.016^2^; 0.022^3^
Imagery craft sector/Other craft sectors vs. WHOQOL-BREF, Environment Domain	0.023^6^
Other craft sectors vs. Main benefit of involvement in the craft sector: Well-being	< 0.001^2^; < 0.001^3^

*p < 0.05. Statistical analysis methods used:

1 Spearman’s Rank Correlation Coefficient: r_s_

2 Chi-square Test: *χ*^2^.

3 Fisher’s Exact Test: FET.

4 Mann–Whitney U Statistic: U.

5 *F*-statistic (used in ANOVA): *F*.

6 *t*-test Statistic: *t*.

## 3 Results

### 3.1 Sociodemographic, health condition, and professional characteristics

Table 1 describes the study variables related to participants’ sociodemographic characteristics and health condition. The sample consisted of 55 individuals, representing 27.36% of the artisans in the territory. Participants had a mean age of 67.49 years (SD 8.02), ranging from 55 to 88 years. More than half were men (60%, *n* = 33).

Most participants were married (85.5%; *n* = 47). The average number of years of education was 6.42 years (SD 3.32). The range of education was from 0 to 19 years, with 43.6% (*n* = 24) of the participants having completed up to 4 years of education and 25.5% (*n* = 14) having completed between 7 and 9 years. Overall, 23.6% (*n* = 13) of the sample had professional training, with 11 different areas reported (*n* = 11). Most participants (60%, *n* = 33) were retired and nearly all (96.4%, *n* = 53) lived in their own house. The mean years of residence in the parish was 56.00 (SD = 19.50, range 3–84 years).

Overall, 89.1% (*n* = 49) of the participants considered their craft activity to be their main occupation. Almost all artisans, 98.2% (*n* = 54) considered that their main source of income was from work in crafts, with 58.2% (*n* = 32) also citing retirement, and only a minority (10.9%, *n* = 6) mentioning other sources of income. Most artisans (63.6%, *n* = 35) reported that their monthly income was equal to or less than the national minimum wage.

All participants had the National Health Service as their health subsystem, and the majority (52.7%, *n* = 29) reported visiting the Health Center every 6 months.

In the past year, nearly all participants (94.5%, *n* = 52) utilized the Health Center, and the majority (61.8%, *n* = 34) also attended specialty consultations. Emergency services were used by 16.4% (*n* = 9) of participants. A minority (7.3%, *n* = 4) reported having been hospitalized, with durations ranging from 1 to 12 days.

Most participants (52.7%, *n* = 29) work in the Imagery sector, followed by 12.7% (*n* = 7) in the Pottery sector, and 9.1% (*n* = 5) in the Wood sector.

The age at which participants started their craft activity ranges from 4 to 63 years, with a median of 12 years. A large proportion (43.6%, *n* = 24) began in childhood (by the age of 10), while only one participant (1.8%) started after the age of 60. Most artisans (87.3%, *n* = 48) have their artistic atelier located at their residence.

On average, artisans dedicate 51.24 h (SD 17.05, range 18–96) per week to their craft activity. The majority of participants (69.1%, *n* = 38) engage in craft activity for more than 40 h per week.

In addition to their activity on weekdays, artisans also engage in their craft on Saturdays (65.5%, *n* = 36), and 30.9% (*n* = 17) reported doing so occasionally on Sundays.

With respect to activities regularly pursued outside their craft work, artisans allocate an average of 10.05 h per week (SD 9.33). The most mentioned were household activities and subsistence farming (45.5%, *n* = 25), physical activity (36.4%, *n* = 20) and sociocultural activities (29.1%, *n* = 16). A total of 16.4% (*n* = 9) of the participants reported not engaging in any regular non-professional activities. Table 2 describes the study variables related to the participants’ professional and artistic sector.

#### 3.1.1 Associations between sociodemographic and professional characteristics

Age positively correlated with the number of years of residence in the parish (*p* < 0.001) and negatively correlated with years of education (*p* < 0.001), the number of weekly hours dedicated to non-professional activities (*p* < 0.001), as well as with the age at which individuals began their activity in the craft sector (*p* < 0.032).

A positive correlation was observed between years of education and the age at which individuals began working in the craft sector (*p* = 0.012) and with the number of weekly hours dedicated to regularly performed non-professional activities (*p* < 0.006).

Women engaged significantly more in household activities during their non-professional time (*p* < 0.001), while they were less likely than men to report involvement in cultural activities (*p* = 0.034). A small difference was observed in the comparison of mean of hours dedicated to craft activities between retirees and non-retirees (M = 50.70, SD = 16.17; M = 52.05, SD = 18.65) which was not statistically significant.

A significant association was also found between non-professional activities and caregiving (*p* = 0.011), indicating that non-retirees are more likely to engage in caregiving activities.

A statistically significant difference was observed in the number of years of residence in the parish between individuals who reported a monthly income equal to or less than the national minimum wage and those with a higher monthly income (*p* = 0.032). Individuals with a lower income have resided in the parish for a longer period (Mean Rank = 31.50) compared to those with a higher income (Mean Rank = 21.88). Although the results indicate that the average age is slightly higher in the lower-income group (M = 68.23, SD = 7.535) compared to the higher-income group (M = 66.20, SD = 8.877), the tests performed did not show a significant difference in variances between the groups.

No statistically significant difference was found in the variable of the number of hours dedicated each week to craft activity between the income groups.

Regarding activities performed outside of craft work, caregiving (*p* = 0.049) and participation in household activities (*p* = 0.025) were more frequently reported by the Imagery sector.

### 3.2 Variables related to health, quality of life, and happiness

Regarding disease status, 81.8% (*n* = 45) of individuals reported having been diagnosed with at least one disease throughout their lifetime. In the frequency analysis, the most commonly reported were one disease (*n* = 22, 40%), two diseases (*n* = 17, 30.9%), and no disease (*n* = 10, 18.2%).

Various diseases were reported (*n* = 23), with osteoarthritis being the most mentioned (18%, *n* = 10), followed by spinal disorders (16.4%, *n* = 9), and bone diseases such as those affecting the hip and knees (12.7%, *n* = 7).

According to the cutoff points of the Patient Health Questionnaire (PHQ-8), 41.8% (*n* = 23) of participants scored the minimum of zero, and the remaining scores were below the cutoff for mild depression, indicating no depression, except for one participant who scored 13, suggesting moderate depression.

The observation of responses on the WHOQOL-BREF quality of life scale showed that, regarding general health perception (Question 2: How satisfied are you with your health?), the majority of artisans (60%, *n* = 33) considered themselves satisfied or very satisfied with their health, 34.5% (*n* = 19) reported feeling neither satisfied nor dissatisfied, and a minority (5.5%, *n* = 3) reported being dissatisfied.

The general perception of the majority of artisans (61.8%, *n* = 34) regarding their quality of life was ‘good’ (Question 1: How would you rate your quality of life?). The transformed scores from the WHOQOL-BREF quality of life assessment instrument showed that the mean score for overall quality of life perception (questions 1 and 2) was 68.64 (SD = 13.58). The psychological domain had the highest average score (M = 84.92, SD = 10.98), followed by the social relationships domain (M = 80.76, SD = 12.61), the environment domain (M = 78.81, SD = 9.48), and the physical domain (M = 78.64, SD = 11.91).

Most artisans (56.4%, *n* = 31) reported feeling “very happy” about their current life (Table 3 describes the objective health indicators and outcomes related to health, quality of life, and happiness).

Regarding the perceived main benefits from craft work (well-being, economic, health, quality of life, social status), well-being was by far the most frequently reported by artisans (74.5%, *n* = 41), followed by economic (32.7%, *n* = 18), health (23.6%, *n* = 13), quality of life (21.8%, *n* = 12), and social status (12.7%, *n* = 7). The analysis of these benefits by craft sector, Imagery versus Pottery, Basketry and Wicker, Iron and Derivatives, Wood, Embroidery, Weaving, and Contemporary Crafts, confirmed that well-being remained the most frequently cited benefit regardless of the sector.

### 3.3 Associations between sociodemographic and professional variables, objective health indicators, and outcomes related to health, quality of life, and happiness

A significant positive correlation was observed between age and the degree of happiness (*n* = 55, r_s_ = 0.312, *p* = 0.020).

The happiness questionnaire revealed that most women (63.6%, *n* = 14) rated themselves as very happy, while 36.4% (*n* = 8) felt good and happy. Among men, 51.5% (*n* = 17) felt very happy, and 45.5% (*n* = 15) felt good and happy.

There was a significant association between gender and the presence of osteoarthritis in the hands and/or fingers [*χ*^2^(1) = 12.731, *p* < 0.001], also verified by Fisher’s exact test (*p* < 0.001), indicating that this condition is more prevalent among women.

Significant differences were observed between genders in two of the main benefits of involvement in the craft sector (well-being, economic, health, quality of life, social status). Women were more likely to perceive the benefit related to quality of life [*χ*^2^ = 4.548; df = 1; *p* = 0.033; Fisher’s Exact Test: *p* = 0.047 (2-sided)], while men were more likely to identify well-being as the primary benefit (*χ*^2^ = 4.615; df = 1; *p* = 0.032).

The overall perception of quality of life and health as measured by the WHOQOL-BREF showed a significant difference based on income (U = 223.500, Z = −2.366, *p* = 0.018). The mean score was higher in the group reporting a higher income (Mean Rank = 34.33) compared to individuals reporting a lower income (Mean Rank = 24.39). A statistically significant difference was also observed in the environmental domain of quality of life between income groups (*F* = 5.157, *p* = 0.027; t = −3.884, *p* < 0.001). The Cohen’s d value is −0.987 (95% CI: −1.563 to −0.402), indicating a large effect, with a higher mean score in the higher-income group (M = 34.95, SD = 2.114) compared to the lower-income group (M = 32.23, SD = 3.059).

The PHQ-8 results indicated that nearly all participants (98.1%) scored between zero and below the minimum cutoff point for depressive symptoms. A significant negative correlation was found between the Environment domain of the WHOQOL-BREF and the PHQ-8 scores (*n* = 55, r _s_ = −0.294, *p* = 0.029), indicating that better conditions in this domain are strongly associated with lower levels of depressive symptoms.

Among the examined variables of the main benefits of craft activities, the Chi-square test revealed a significant difference in the health-related benefit between the groups with and without diseases [*χ*^2^(1) = 4.706, *p* = 0.030].

Significant differences were examined in quality of life and health status variables between participants who reported one or more illnesses (*n* = 45) and those who did not report any illnesses (*n* = 10). The results indicate that the WHOQOL-BREF General Quality of Life variable showed a statistically significant negative difference (U = 123.000, Z = −2.380, *p* = 0.017) between the groups. The general perception of quality of life and health status was higher in the group that did not report any illnesses (Mean Rank: 38.20) compared to the group that reported illnesses (Mean Rank: 25.73).

Regarding the average hours spent weekly on craft activities between the group that reported one or more diseases and the group without reported diseases, the average did not differ significantly (*p* > 0.05).

Additionally, non-professional activities are not strongly associated with the presence or absence of diseases.

Associations and statistically significant differences were investigated between the Imagery sector (*n* = 29) and other craft sectors, including Pottery, Basketry and Wicker, Iron and Derivatives, Wood, Embroidery, Weaving, and Contemporary Crafts (*n* = 26).

Significant differences were observed in the variable concerning the main benefits of craft work (well-being, economic, health, quality of life, social status). Chi-square and Fisher’s Exact Test results revealed that participants in the Imagery sector reported health benefits more frequently [*χ*^2^ = 6.945; df = 1; *p* = 0.008; Fisher’s Exact Test: *p* = 0.011 (2-sided)], and quality of life benefits [*χ*^2^ = 5.768; df = 1; *p* = 0.016; Fisher’s Exact Test: *p* = 0.022 (2-sided)]. Conversely, well-being was reported more frequently by participants from other sectors [*χ*^2^ = 12.134; df = 1; *p* < 0.001; Fisher’s Exact Test: p < 0.001 (2-sided)].

There is a statistically significant difference [*t*(53) = −2.346, *p* = 0.023] in the environment domain of quality of life between the Imagery sector and the other sectors (Pottery, Embroidery, Weaving, Iron and Derivatives, Wood, Basketry and Wicker, Contemporary Crafts). The second group reported a higher average score (M = 34.19, SD = 2.367) compared to the Imagery sector (M = 32.34, SD = 3.330), with a mean difference of −1.847, 95% CI [−3.427, −0.268]. The Cohen’s d value of −0.634 indicates a medium effect size (Table 4 summarizes the significant associations between sociodemographic and professional variables, objective health indicators, and outcomes related to health, quality of life, and happiness).

In the descriptive analysis, the psychological domain of quality of life remained the highest rated among both the Imagery sector (M 83.48, SD 11.54) and the other craft sectors (M 86.54, SD 10.29). For the other domains, the second highest rating in the Imagery sector was the social relationships domain (M 80.46, SD 12.25), whereas in the other sectors it was the environment domain (M 81.85, SD 7.39), followed by social relationships (M 81.09, SD 13.24). The least rated domain in the other sectors was the physical domain (M 78.57, SD 10.49), while in the Imagery sector, the least rated domain was the environment domain (M 76.08, SD 10.40).

## 4 Discussion

This study differs from previous research in several important ways and provides relevant scientific evidence on the perception of health status, quality of life, and happiness among older individuals engaged in crafting, based on a sample of professional artisans from a UNESCO Creative City of Crafts and Folk Art.

All study variables were tested to examine significant associations between sociodemographic characteristics, professional activity-related variables, and measures of health, quality of life, and happiness.

Several significant correlations and differences were observed between the sociodemographic and psychosocial variables of the participating artisans, reflecting the association of factors such as age, education, and professional craft activity with different aspects of the participants’ health status, quality of life and happiness. The results showed a trend in which older artisans reported greater happiness, had fewer years of formal education on average, spent fewer weekly hours on non-craft activities, and began their craft work at a younger age, reflecting generational differences in work trends. Like in other professions, people in the past generally started their craft activity earlier than they do today. It was found that individuals with more years of education started their craft activity later, which suggests an association with the evolution of mandatory education over time. Furthermore, these younger individuals also tend to dedicate more weekly hours to other activities during their non-professional time.

The results also showed that older artisans remained in the same locality as they aged, which may indicate residential stability, a connection to the place, family, and local community, and a stronger bond with the sociocultural territory.

On the other hand, the significant positive correlation found between age and the level of happiness suggests that older participants experience higher levels of happiness. This finding appears to be aligned with previous research indicating that happiness tends to increase after the age of 50 (Becker and Trautmann, 2022). However, the U-shaped pattern of happiness over the lifespan remains a controversial finding in the literature. While some studies support this trend, others suggest that happiness can be shaped through practice (Esch, 2022) or that happiness is malleable and can be enhanced (Steptoe, 2019).

Among the benefits of engaging in crafting, it was observed that gender may influence the perception of certain benefits. Women placed a higher value on quality of life, while men valued well-being more. For activities regularly performed during leisure time, women showed a significantly greater tendency to engage in domestic activities and less in cultural activities compared to men. In light of this result, a significant trend or difference in the time dedicated to crafting between men and women was anticipated, but this was not observed. This may indicate that a lack of leisure time beyond professional activities might limit women’s participation in activities other than domestic chores. This finding is consistent with previous studies (Cruz, 2004; Fernandes, 2024) examining gender issues in this field, which note that while both women and men worked with clay, there was a division of labor, with women alternating between figurine crafting and domestic responsibilities, including child care. These results suggest that this dynamic may still be present.

The data shows that most individuals, both female and male, reported feeling very happy. Previous studies have provided relevant insights into the individual and social importance of everyday creativity related to craft-based activities for promoting well-being and overall health (e.g., Tzanidaki and Reynolds, 2011; Kenning, 2015; Pöllänen and Weissmann-Hanski, 2020). The results of this study expand the scope of diversity in the analysis by including additional sectors related to craft.

When comparing retirees and non-retirees, a difference in the number of hours dedicated to craft activity was anticipated; however, the results showed no statistically significant differences in the weekly hours spent on the activity. While age is a characteristic that clearly differentiates the two groups, both retirees and non-retirees are intensively engaged in the activity.

Furthermore, the similar amount of time dedicated to craft activities, regardless of income, appears to reflect intrinsic motivations, such as a passion for their art and personal satisfaction. Previous studies have highlighted that various factors contribute to the meaning of work (Rosso et al., 2010). Harmonious passion has a positive effect on psychological well-being, physical health, positive relationships, and high-level performance. Therefore, having a harmonious passion for an activity can greatly contribute to living a meaningful life (Vallerand, 2012). Although the situation regarding income has not revealed a difference in the number of hours dedicated to craft activity, as both groups, those with a monthly income equal to or below the national minimum wage and those with an income above it, showed similar levels of dedication to the activity, income appears to significantly influence the perception of quality of life and health, as well as the environment domain. Indeed, the higher-income group perceives their quality of life more positively compared to the lower-income group. This suggests that, for these artisans, while income does not appear to affect their dedication to craft activity, it does influence other factors related to their overall perception of quality of life and health. Specifically, aspects of the WHOQOL-BREF environment domain (such as opportunities for acquiring new information and skills, participation and/or opportunities for recreation and leisure, physical environment, transportation), are perceived differently by those with lower income.

Previous studies assessing quality of life among older adults who work and those who do not have demonstrated that staying in work, in addition to providing health benefits, contributes to autonomy and improves quality of life. It was observed that older adults who do not work show lower averages in the environment domain, which are associated with quality of life impairments due to factors such as income (Costa et al., 2018). These findings emphasize the relevance of income as an influential factor on quality of life among older populations, as demonstrated by the results of the present study, conducted with a population professionally dedicated to craftsmanship.

These results highlight the importance of public policies that promote income improvement as a strategy to enhance quality of life for all artisans. The environment or social context are among the factors that influence the meaning attributed to work (Rosso et al., 2010). In this study, it is particularly important to highlight the favorable context for the valuation of craftsmanship in the analyzed territory. This is a region with a long tradition of craftsmanship and folk art. In this territory, the municipality has been implementing public policies and initiatives that value craftsmanship, such as certified craft productions, and it is recognized by UNESCO as a Creative City of Crafts and Folk Art (Município de Barcelos, 2023; UNESCO, 2024).

This set of factors appears to play a significant role in creating a context conducive to the development of craftsmanship, functioning as a socially and culturally protective environment for this population dedicated to craftsmanship. This environment also seems to help explain some of the positive results observed, such as the perception of happiness associated with aging, as indicated by the positive correlation between age and happiness, although further studies should be conducted.

These results align with studies that, when analyzing how older adults experience and describe the fundamental pillars of active aging suggested by the WHO, describe health as the absence of barriers to daily living, security as manageable living conditions, participation as meaningful activities, highlighted perspectives related to daily life experiences and local culture, add motivations for engagement linked to local traditions, and clarify that appropriate participation creates a sense of belonging in society (Wongsala et al., 2021).

Our results showed an almost complete absence of depressive symptoms, which is a positive indicator of the mental health status of these older professional artisans, considering the findings from the latest National Health Survey data conducted in Portugal using the PHQ-8 methodology. This national survey concluded that approximately 60% of individuals showed mild symptoms, and 40.2% exhibited severe depressive symptoms. The study also found that the prevalence was more pronounced among the older population (Instituto Nacional de Estatística, 2020). This stresses further the good condition of our population, comprising individuals aged 55 and older, of whom 41.8% had a score of zero, and the remaining being below the cut-off score for depression with the exception of one participant.

The significant negative correlation identified between the environment domain of the WHOQOL-BREF and the PHQ-8 shows that better conditions in the environment domain are associated with lower levels of depressive symptoms. This indicates that positive environmental conditions may have a significant impact on individuals’ mental and emotional health, and that good mental health may also influence a better perception of environmental conditions.

The findings support larger-scale studies conducted in Portugal that analyzed active aging in later life and identified the relevance of psychological aspects in active aging, such as the absence of psychological distress, the presence of happiness and optimism, and quality of life, to enable active engagement with life despite common health issues in advanced age (Paúl et al., 2017).

The most reported illnesses in this study align with findings from other studies conducted in Portugal. The conclusions described in the most recent National Health Survey regarding chronic conditions self-reported by the Portuguese population include osteoarthritis, lower back pain or other chronic back problems, and cervical pain. That study concluded that these conditions affect women more than men (Instituto Nacional de Estatística, 2020). Consistent with these findings, our study shows more women reporting the most common diseases among participants (e.g., osteoarthritis).

It was anticipated that there would be an association between the group with reported diseases and a lower number of hours dedicated to craft activities. Although this group shows a lower perception of overall quality of life and the physical domain, the results regarding the number of hours dedicated reveal that the dedication to craft activities remains strong. These data suggest that the reported diseases do not have a significant impact on the amount of time participants devote to their craft activities. This behavior may reflect an effort and adaptation to maintain involvement in the craft, even in the face of some physical difficulty or limitation.

The study also revealed a significant difference between the group with reported illnesses and the group without reported illnesses regarding the perceived main benefits of craft activities, particularly in relation to the health benefit. The fact that the group without reported illnesses valued good health as a benefit of these activities suggests that, for these individuals, participation in craft activities may play an important role in the perception of health improvement or maintenance. Furthermore, it was also in the group without reported illnesses that a better overall perception of quality of life and health status was observed on the WHOQOL-BREF scale compared to the group that reported illnesses. The involvement of older individuals in these activities may be associated with a positive impact on health, aligned with results from previous studies (Kenning, 2015; Pöllänen and Weissmann-Hanski, 2020).

Most comparisons between participants from the Imagery sector and those from other sectors did not indicate statistically significant differences, suggesting similarities between the participants in the groups in characteristics such as age, education level, age at the start of their craft career, average number of hours per week dedicated to craft activity, length of residence in the parish, as well as in the Patient Health Questionnaire (PHQ-8) and WHOQOL-BREF scales, particularly in the physical, psychological, social relationships domains, and in the measures of overall quality of life and general health perception. The degree of happiness of artisans when considering their current life also indicates similarity between both groups. Only one statistically significant difference was identified between the sectors in the environment domain of the WHOQOL-BREF quality of life scale. The group from the Pottery, Embroidery, Weaving, Iron and Derivatives, Wood, Basketry and Wicker, and Contemporary Crafts sectors showed a higher average, suggesting that these participants have a more positive perception of the facets included in the environment domain compared to the Imagery sector group. In the quality of life scale, the environment domain includes facets such as: physical safety; home environment; economic resources; health and social care (availability and quality); opportunities to acquire new information and skills; participation in and/or opportunities for recreation and leisure; physical environment (pollution/noise/traffic/climate); and transportation. The observed difference may suggest that the Imagery sector might involve specific characteristics or challenges that affect participants’ perceptions of their environment, such as opportunities for acquiring new information and skills.

Additionally, significant differences were observed in the perceived benefits of craft activity (well-being, economic benefits, health, quality of life, social status) among the craft sectors, particularly in health, quality of life, and well-being. Participants from the Imagery sector highlighted more benefits related to health and quality of life, while well-being was more frequently reported by participants from other sectors. These findings align with studies that concluded that active engagement in participatory arts enhances quality of life, self-assessed health, and life meaning (Ho et al., 2019).

Additionally, the results are consistent with studies that used the WHOQOL-BREF scale to assess quality of life, which included older adults who remain active in the workforce. Previous studies identified higher mean scores in most of the scale domains, particularly in the psychological domain (M = 70.0; SD = 11.8), suggesting that work is an important factor for quality of life among older adults (Costa et al., 2018). In the present study, conducted with older professional artisans, a similar pattern was observed, with an even more pronounced focus on the psychological domain (M = 84.92; SD = 10.98). Moreover, the results were higher across all domains of the scale. High scores were also observed in the assessment of happiness, as well as results indicating the near absence of depressive symptoms, as reported by the participants in the depression symptomatology assessment. These findings are particularly relevant when compared to other studies conducted in the same country using the same scale (Instituto Nacional de Estatística, 2020).

The results observed in this sample of individuals whose daily lives are primarily filled with craft-related activities suggest consistency with findings from various international studies (Zhang et al., 2018; Pöllänen and Weissmann-Hanski, 2020; Keyes et al., 2024). Studies involving artisans up to the age of 88 have shown that crafting improves participants’ psychological well-being in various ways and can lead to a richer, more purposeful life through self-fulfillment, excellence in craftsmanship, and a sense of belonging (e.g., Pöllänen and Weissmann-Hanski, 2020). Furthermore, other research has verified that a sense of meaning in life is associated with happiness and can contribute to health and utilization of health services among older adults (e.g., Zhang et al., 2018). Additionally, studies have demonstrated that engaging in creating arts and crafting significantly predicts increased life satisfaction, a sense that life is worthwhile and happiness (e.g., Keyes et al., 2024).

Unlike most studies, this one focuses on activities within the craft and folk art sector, conducted in the context of spontaneous professional engagement by older adults (e.g., Chacur et al., 2022). It contributes to the recognition of these individuals by highlighting their creativity and their role as an asset in the economic and sociocultural dynamics of their families and communities.

These results may be related to the type of activity performed by the professional artisans and the meanings attributed to these activities (Wrzesniewski and Dutton, 2001; Rosso et al., 2010; Vallerand, 2012), which can have positive implications for quality of life and psychological health in older adults. Participation in creative and culturally meaningful activities, such as crafts and folk art, can be seen as an expression of generativity, a central concept in Erikson’s (1950) theory. It argues that the continuity of psychological development throughout life is essential, and the stage of old age is characterized by the search for integrity rather than despair. Work and activities that promote active engagement, such as crafts, allow older individuals to experience a dynamic balance of opposites, a fundamental principle for well-being and psychological health in old age (McAdams and de St Aubin, 1992; McAdams et al., 1993; Kivnick and Wells, 2014). These findings suggest that older professional artisans may be achieving integrity, a state of acceptance and satisfaction with their lived life, which is crucial for their psychological health. However, additional studies are needed to confirm these findings and further explore these relationships.

### 4.1 Study contributions and future research

No previous studies have identified the contribution of craft and folk art to the health, quality of life and happiness among older professional artisans. To the best of our knowledge, this study represents an original and significant contribution to the field of aging research. It addresses gaps that have been identified in the literature by expanding the scope of artistic activities considered, such as arts and crafts (Fraser et al., 2015; Chacur et al., 2022). It provides innovative information by presenting data that explores various craft sectors and diverse variables related to the health status, quality of life and happiness of older professional artisans, such as gender, retirement status, income, time devoted to craft activities, extra-professional activities, and health-related factors. The diversity of this study is a significant strength, both in terms of representing various activities and in including both men and women, which also broadens the scope of previous studies (e.g., Tzanidaki and Reynolds, 2011; Kenning, 2015; Pöllänen and Weissmann-Hanski, 2020).

While there is a wealth of research on older adults’ participation in artistic activities, other activities, such as arts and crafts, are underrepresented (Chacur et al., 2022). Although intervention studies analyzing artistic activities are common, research on spontaneous artistic activities that occur in natural contexts (i.e., not resulting from interventions) remains sparse (Tzanidaki and Reynolds, 2011; Chacur et al., 2022). The study contributes to this knowledge and stands out for the variety of variables collected to characterize the perceptions of older professional artisans. It includes a broad set of contextual and psychosocial data, both modifiable and non-modifiable (sociodemographic variables, variables related to artistic activity and health, multidimensional quality of life assessment, depression symptomatology evaluation, and happiness assessment) to provide a detailed and comprehensive analysis of the sociodemographic profiles and the role of crafting in the perceptions of health status, quality of life, and happiness among older professional artisans.

This study adds to previous research highlighting the need for investigation into the role of active aging in quality of life and health (Hijas-Gómez et al., 2020), by demonstrating the benefits of remaining active in old age.

However, there are some study limitations, namely the inexistence of a control group of old people in the same city that have other occupations (e.g., agriculture), and the study of a convenience sample, which may affect the generalizability of the findings. Nevertheless, it must be noted that the sample represents almost 30% of the artisans in the territory, providing a useful insight into this population. To explore in depth the perceptions of artisans related to health and quality of life, as well as associations between craft sectors, it would also be useful to understand contextual factors influencing perceptions of health status, quality of life, and happiness. Other factors as personal motivation, family cohesion, and the legacy of craftsmanship, which may be related to commitment to artistic activity would be relevant to the comprehension of the role of crafts in the selected outcomes.

Future research would benefit from a larger sample and longitudinal studies, both quantitative and qualitative, also involving younger professional artisans from different sectors. Increasing the sample size would allow for comparative analyses between groups of different ages, enabling the exploration of associations that could expand the understanding of the relationships between sociodemographic characteristics, professional sector variables, and perceptions of health, quality of life, and happiness. In future studies, it is recommended to incorporate a multidimensional happiness assessment tool, which would allow for a more comprehensive analysis of different domains of subjective well-being. Such studies, could provide relevant scientific insights and help create more targeted programs that address specific needs, allowing for the exploration of observed associations and providing more robust data.

Investigating how working conditions can be adjusted to better accommodate the needs of older individuals with physical conditions and health issues common in advanced ages could provide valuable insights to science. Studies that delve into associations between craft sectors and examine the implications of health conditions on productivity and well-being, and that explore how health-related situations affect the subjective experience of working in craft activities, could contribute additional information.

Despite its limitations, the findings from this study serve as important starting points for future empirical research, enabling a critical understanding of the relationship between professional craft and folk art activities among older artisans and their perception of health status, quality of life, and happiness, as well as for comparative studies between different UNESCO creative territories.

Recent studies on active aging (Shahla et al., 2023) conclude that researchers, when designing active aging programs, should pay special attention to factors such as gender and cultural background of older adults, as these are significant considerations in program design. Our study adds valuable information to active aging programs by considering these and other factors, based on activities related to the sociocultural identity of the participants (UNESCO, 2024).

The information emerging from this study can be valuable in guiding the development of targeted programs aimed at enhancing the health and quality of life of professional artisans. Initiatives focusing on occupational health promotion, along with educational actions on safe work practices, can benefit artisans by reducing the incidence of work-related illnesses. These measures can improve quality of life while fostering a safe and healthy work environment.

The development of health and wellness programs could be considered. Actions focusing on correct posture techniques, proper tool usage, and practices that minimize physical stress can help reduce the prevalence of occupational diseases. Incorporating breaks and appropriate physical exercises can aid in injury prevention, thereby mitigating the impact of these conditions on health. Activities focused on creating appealing opportunities for older adults, which encourage participation in physical, educational, and sociocultural activities tailored to their needs and health conditions, can support the balance between professional and personal life and contribute to improving the health and quality of life for artisans. Additionally, these results highlight positive concepts associated with aging. They align with the current global agenda for promoting a more equitable, inclusive, and sustainable society, and contribute valuable information to the fight against ageism (Nações Unidas, 2018; World Health Organization, 2020, 2021).

Finally, at a time when countries are increasingly seeking innovative and cost-effective methods to improve the health and well-being trajectories of rapidly aging populations, and where there is a growing emphasis on measures of quality of life, well-being, and happiness as decision-making metrics for guiding public policies (Helliwell et al., 2023; United Nations, 2023), this study, by expanding the professional involvement of older adults and the artistic activities within the craft and folk art sector, provides new insights and avenues for research in the scientific community.

### 4.2 Conclusion

The study aligns with national and international strategies for active and healthy aging (United Nations, 2020; World Health Organization, 2020, 2021; United Nations, 2023; Presidência do Conselho de Ministros, 2024).

A strong dedication to craft activities among older individuals was observed in this study. The findings highlight significant participation of older adults in these crafts, indicating that their involvement has continued over time, as supported by the literature on certain crafts in this region. For example, in activities related to clay figures: “it is the older adults who are the creators of this realism, and they are the only ones who make everything” (Costa, 1991, n.p.; Costa, 2024, p. 145).

The results show a trend where older artisans reported higher levels of happiness. This finding aligns with previous studies that indicate an increase in happiness after the age of 50 (Becker and Trautmann, 2022). However, this finding should be interpreted with caution, considering the ongoing debate in the literature regarding the life-course pattern of happiness (Steptoe, 2019; Becker and Trautmann, 2022; Esch, 2022). Further research is needed on this topic, employing multidimensional instruments for the assessment of happiness. This study suggests an almost complete absence of depressive symptoms, which is a positive indicator of the health status of these older professional artisans, especially when considering the high prevalence of depression in old people reported in the most recent National Health Survey conducted in Portugal (Instituto Nacional de Estatística, 2020).

In this study, the WHOQOL-BREF quality of life instrument showed a significant association between the environment domain and different craft activity sectors between the Imagery sector and the sectors of Pottery, Embroidery, Weaving, Iron and Derivatives, Wood, Basketry and Wicker, and Contemporary Crafts, with the latter group reporting a higher average score.

The associations observed in the environment domain of the quality of life scale may indicate a favorable context for older adults (World Health Organization, 2007).

An association was found between the different craft sectors and the perceived benefits of the activity, specifically in terms of health, quality of life, and well-being. Although these benefits were perceived differently across sectors, a larger number of participants from the Imagery sector considered health and quality of life as the primary benefits of their activity, whereas artisans from other sectors emphasized well-being. The artisans’ perceptions regarding economic benefits and social status appeared to be quite homogeneous across the craft sectors. In the analysis of health-related variables, it was observed that the health benefit was more highly valued, which may suggest that craft-related activities play a therapeutic or supportive role. On the other hand, economic benefits and social status did not show the same relevance for these artisans.

Considering the high regard for the work of artisans in this region, both nationally and internationally (e.g., Fernandes, 2005; Costa et al., 2024; UNESCO, 2024) a significant association between craft sectors and the perception of social status as a benefit was anticipated. Curiously, this association was not observed. Instead, well-being, health, and quality of life emerged as the more prominent perceived benefits, as confirmed by the results, an interesting conclusion from our study.

This highlights the importance of the active aging and development strategies to help people remain engaged with life (Paúl et al., 2017).

The results of this study can contribute to informed decision-making regarding active and healthy aging, promoting the health, quality of life, and happiness of older adults. This is relevant for both healthcare professionals and policymakers involved in developing public policies focused on the craft and folk art sector.

## Data Availability

The raw data supporting the conclusions of this article will be made available by the authors, without undue reservation.
